# Supplementary material for: Of mice and men: Dendritic architecture differentiates human from mouse neuronal networks
Source: iScience. 2025 Jun 18;28(7):112928. doi: 10.1016/j.isci.2025.112928 (PMC12274884; doi:10.1016/j.isci.2025.112928)
Supplement: Document S1. Figures S1–S16 and Tables S1–S5 [file mmc1.pdf]

iScience, Volume *28*

Supplemental information

Of mice and men: Dendritic architecture differentiates human from mouse neuronal networks

Lida Kanari, Ying Shi, Alexis Arnaudon, Natalí Barros-Zulaica, Ruth Benavides-Piccione, Jay S. Coggan, Javier DeFelipe, Kathryn Hess, Huib D. Mansvelder, Eline J. Mertens, Julie Meystre, Rodrigo de Campos Perin, Maurizio Pezzoli, Roy Thomas Daniel, Ron Stoop, Idan Segev, Henry Markram, and Christiaan P.J. de Kock

Supplemental Information

Networks	Human (II)	Human (EE)	Human	Mouse (II)	Mouse (EE)	Mouse
Nodes	4503	10621	15124	2724	14377	17101
Edges	431393	5717240	9661185	78766	10241482	11797379
Density	0.0213	0.0507	0.0422	0.0106	0.0496	0.0403
In-degree (max)	582	2070	3030	107	1377	1453
In-degree (mean)	95.80	538.30	638.80	28.92	712.35	689.86
Out-degree (max)	413	1600	2514	98	1343	1403
Out-degree (mean)	95.80	538.30	638.80	28.92	712.35	689.86
Max dim	8	12	12	6	9	9
Peak dim	3	5	5	2	4	4
Simplices peak (log)	16.02	24.62	25.55	12.10	23.58	23.67
Simplices sum (log)	16.75	25.49	26.40	12.67	24.23	24.33
Complexity	19.87	27.60	28.72	16.65	26.58	26.88

Table S 1: Structural properties of networks for excitatory, inhibitory, and full networks. II: connections between inhibitory nodes, EE: connections between excitatory nodes for human and mouse.

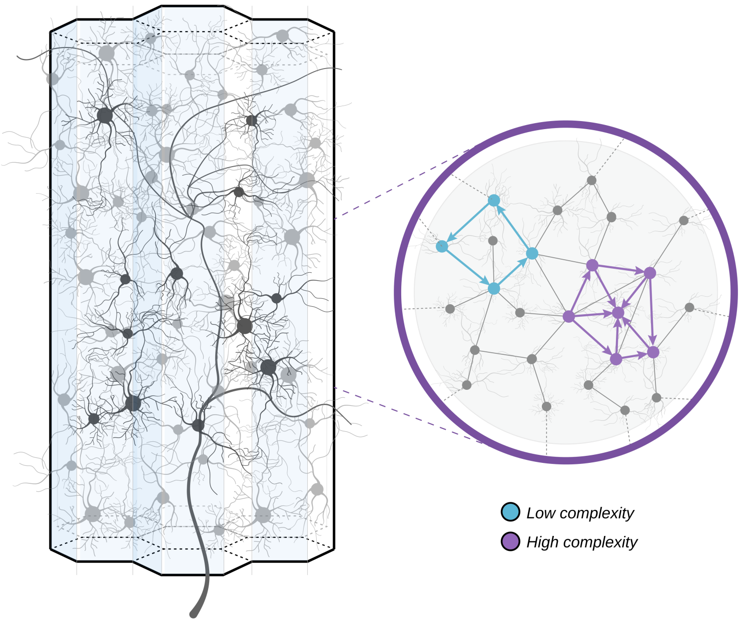

Figure S 1: **Illustration of topological complexity.** Examples of low complexity (blue) for a cycle and high complexity (purple) for a 6-node simplex with one source and one sink.

	HL23PC	HL23BC	ML23PC	ML23BC
Num sections	60.73	74.64	46.74	45.66
Num bifurcations	27.15	34.27	19.34	20.70
Num leaves	33.13	40.18	26.84	24.95
Max radial distance	301.25	383.63	208.09	264.15
Total length	5644.02	6431.81	2952.41	2756.53
Section length	91.06	102.09	62.88	62.35
Mean radii	0.32	0.32	0.34	0.31
Section length (termination)	143.72	154.02	89.40	82.35
Section length (bifurcation)	24.61	29.38	25.65	36.84
Branch orders	5.07	6.25	4.53	5.64
Path distances	141.57	168.20	102.52	154.12
Radial distances	109.94	124.07	79.62	120.99
Section volume	35.44	47.07	24.05	23.37
Section areas	183.12	215.30	133.69	122.12

Table S 2: Average morphometrics for dendritic reconstructions of layers 2 and 3 for mouse (M) and human (H) pyramidal cells (PC) and basket cells (BC).

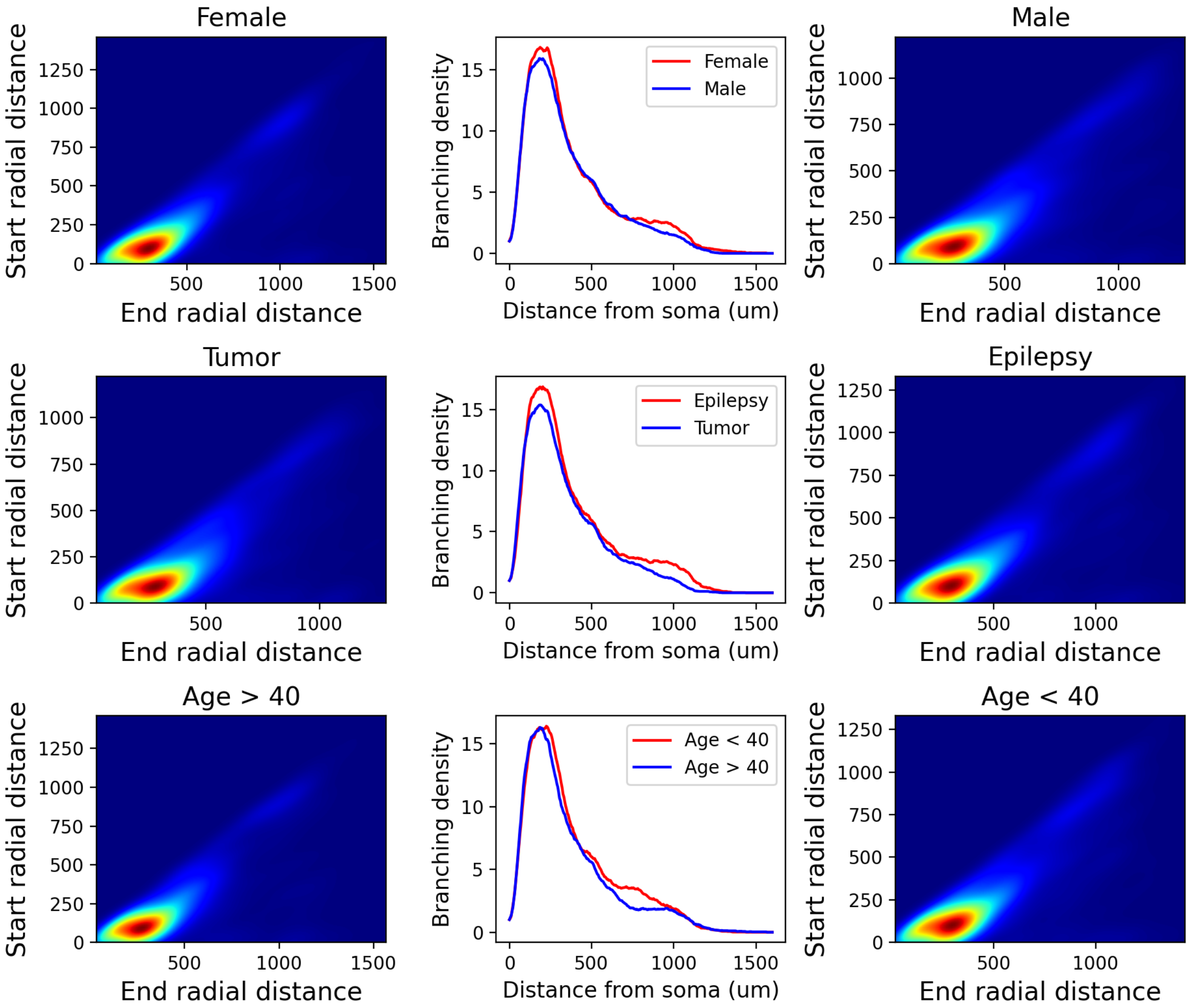

Figure S 2: **Analysis of morphological bias for sex, condition, and age.** Average persistence images of female patients are compared to male patients and their relative average branching density (top). Average persistence images of tumor patients are compared to epilepsy patients and their relative average branching density (center). Average persistence images for ages < 40 patients are compared to patients of > 40 and their relative average branching density (bottom).

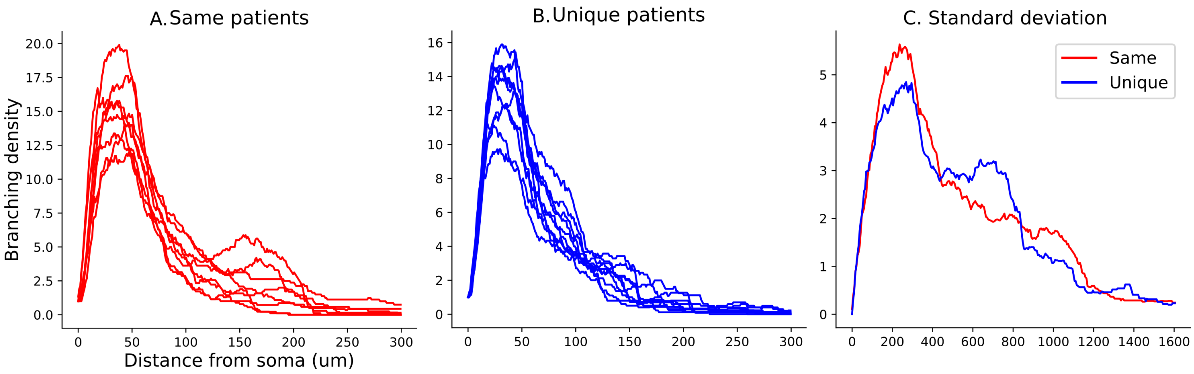

Figure S 3: **Intra versus inter-patient variance.** The average branching density of neurons (A) from the same patients ($7-10$ neurons) is compared to the average branching density of 10 randomly chosen neurons from unique patients (B). The standard deviation of branching density for neurons from the same patient is lower than the standard deviation from neurons of unique patients (C).

	HL23PC	ML23PC
Num sections	54.01	36.21
Num bifurcations	26.04	16.76
Num leaves	27.67	18.92
Max radial distance	692.36	349.13
Total length	5957.94	2523.44
Section length	109.24	70.66
Mean radii	0.34	0.38
Section length (termination)	160.03	93.67
Section length (bifurcation)	55.84	46.22
Branch orders	11.84	8.18
Path distances	291.68	227.77
Radial distances	240.83	185.69
Section volume	51.72	35.10
Section areas	236.73	167.18

Table S 3: Average morphometrics for apical reconstructions of layers 2 and 3 for mouse (M) and human (H) pyramidal cells (PC).

	HL23PC	HL23BC	ML23PC	ML23BC
Num sections	82.26	247.91	161.16	307.86
Num bifurcations	40.46	122.89	80.08	152.61
Num leaves	41.68	124.64	81.08	154.70
Max radial distance	1153.26	646.92	970.59	354.59
Total length	12090.53	17742.66	14906.03	8885.74
Section length	140.31	76.79	106.42	29.97
Mean radii	0.06	0.11	0.08	0.13
Section length (termination)	193.98	93.69	109.75	36.60
Section length (bifurcation)	80.21	59.42	102.84	23.31
Branch orders	11.84	18.29	13.29	18.86
Path distances	568.13	488.25	521.98	204.22
Radial distances	283.59	212.66	273.34	90.35
Section volume	4.5	4.34	2.13	1.82
Section area	59.4	56.73	51.98	23.97

Table S 4: Average morphometrics for axonal reconstructions of layers 2 and 3 for mouse (M) and human (H) pyramidal cells (PC) and basket cells (BC).

	HL23PC	HL23BC	ML23PC	ML23BC
Soma volume	3184.75	2371.79	1522.11	1608.81
Soma surface area	868.05	814.38	630.33	619.7
Soma radius	7.71	7.85	7.02	6.77
Max radial dist	882.71	568.78	1073.61	409.26
Mean width	731.72	793.39	1189.56	391.73
Mean height	1148.49	838.69	1206.9	631.94

Table S 5: Average morphometrics for neuronal reconstructions of layers 2 and 3 for mouse (M) and human (H) pyramidal cells (PC) and basket cells (BC).

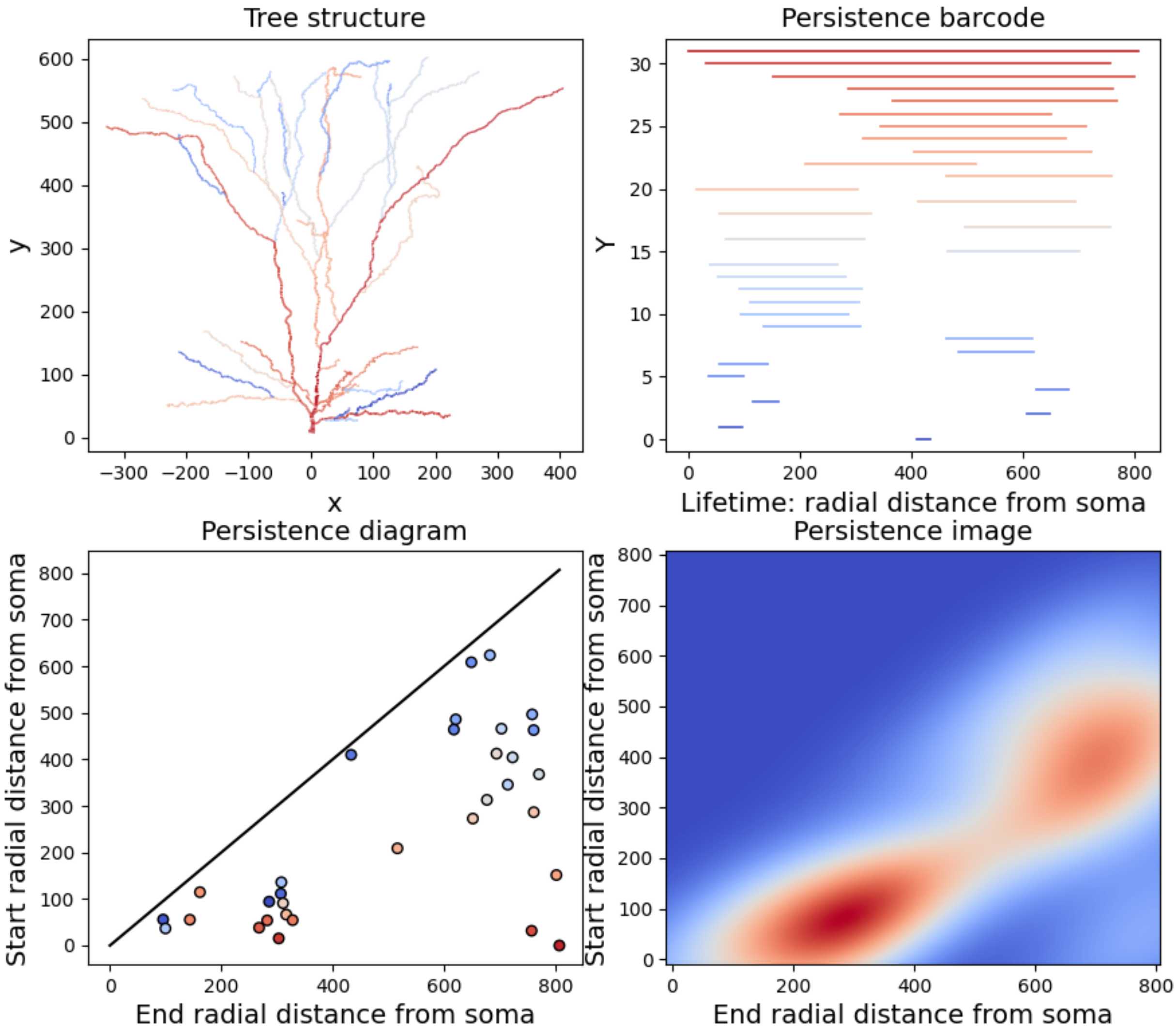

Figure S 4: **Topological morphology descriptor of an exemplar human layer 2 - 3 pyramidal cell apical dendrite.** A. Apical dendrite, color-coded according to persistence components as illustrated in B. B. Persistence barcode, colormap from largest (red) to smallest branches (blue). C. Persistence diagram with the same color-code. D. Persistence image indicating areas of high density of branches (red) at different path distances from the soma (0,0).

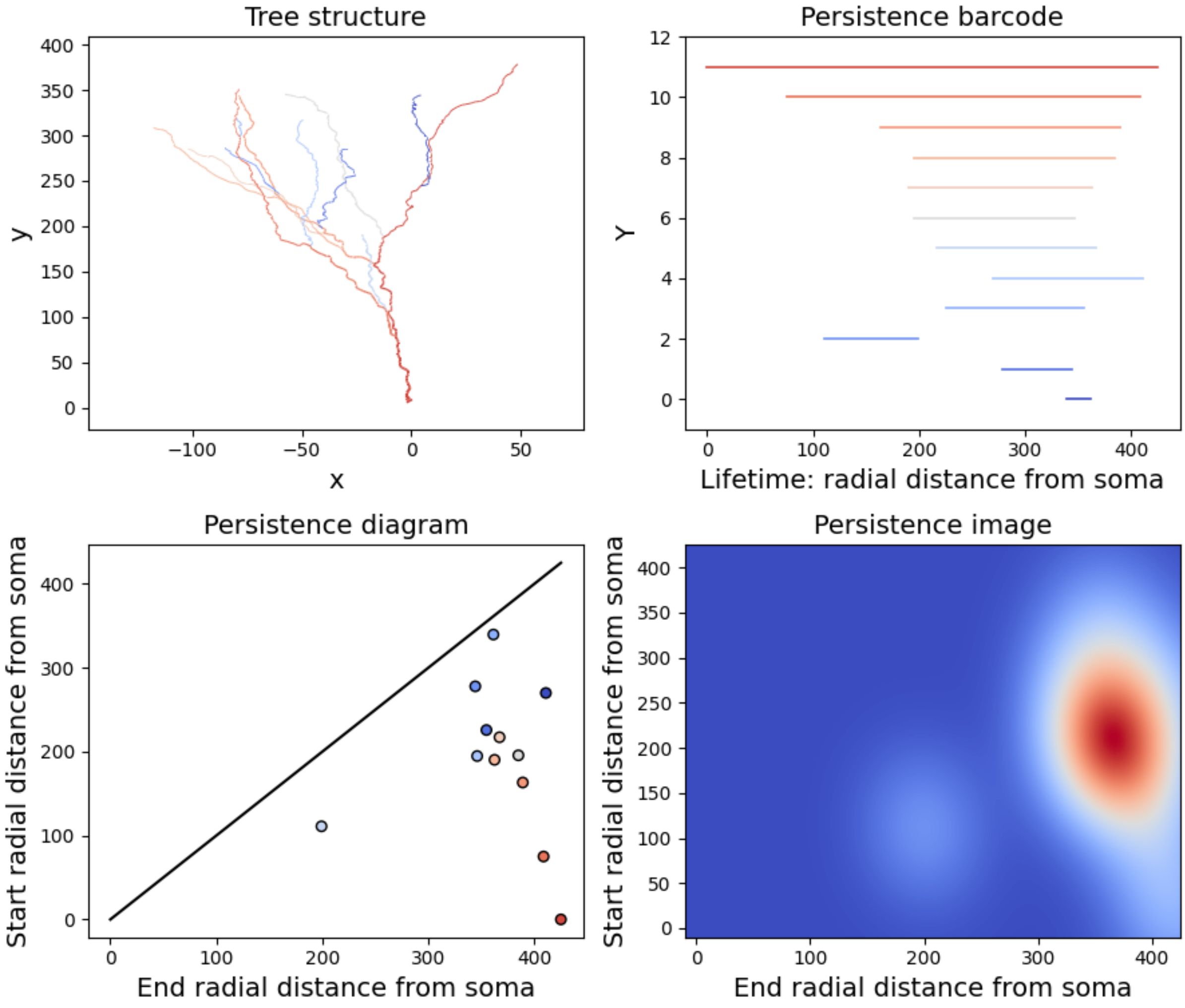

Figure S 5: **Topological morphology descriptor of an exemplar mouse layer 2 - 3 pyramidal cell apical dendrite.** A. Apical dendrite, color-coded according to persistence components as illustrated in B. B. Persistence barcode, colormap from largest (red) to smallest branches (blue). C. Persistence diagram with the same color code. D. Persistence image indicating areas of high density of branches (red) at different path distances from the soma (0,0).

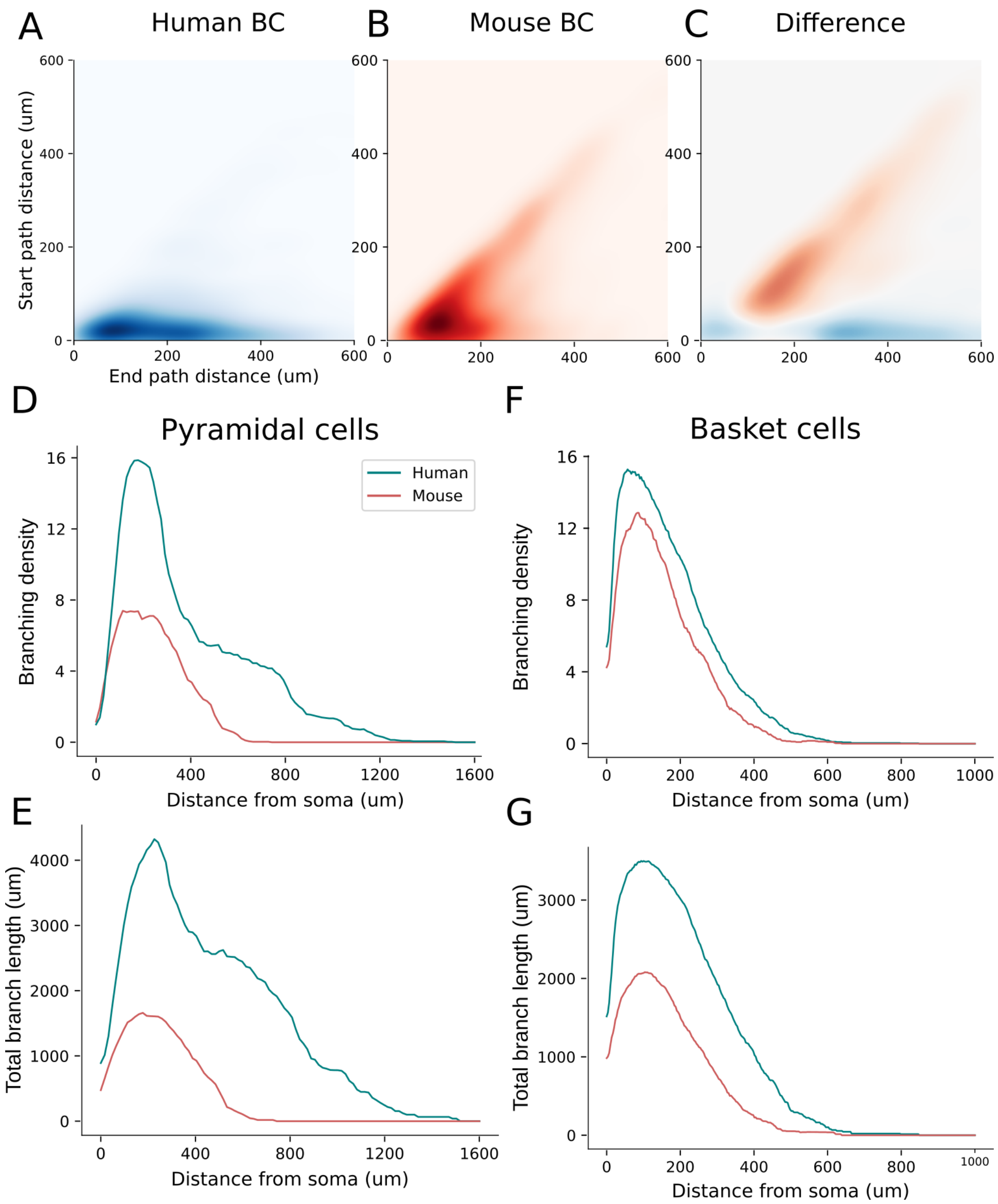

Figure S 6: **Topological analysis of mouse and human interneurons from cortical layers 2 and 3.** A. Average persistence images for populations of human cells. B. Average persistence images for populations of mouse cells. C. Average difference between the persistence images of human (blue) and mouse (red) cells. The comparison of the average topological properties between pyramidal cells (D-E) and basket cells (F-G) of human (teal) and mouse (red) morphologies shows that branching density and lengths are significantly larger in pyramidal cells (D, E). The branching density is only marginally larger in basket cells (F), but branch lengths are significantly larger in human basket cells (G).

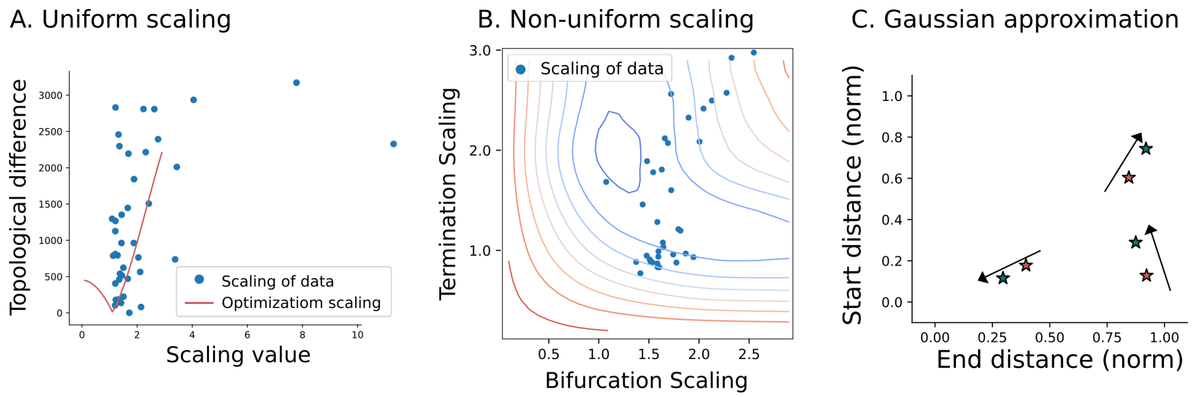

Figure S 7: **Optimization of scaling properties.** A. Uniform scaling (red curve) compared to experimental data (blue points). B. Non-uniform scaling (contour) compared to experimental data (blue points). C. Persistence diagrams represent the start and end radial distances of branches from the soma. Gaussian kernels (3 centers) approximate the density of topological branching within the persistence diagrams of the two species (human: teal stars, mouse: red stars). The optimal transformations of the three Gaussian kernels to convert the normalized persistence diagrams from mouse to human are not aligned, indicating the absence of a consistent transformation between the species.

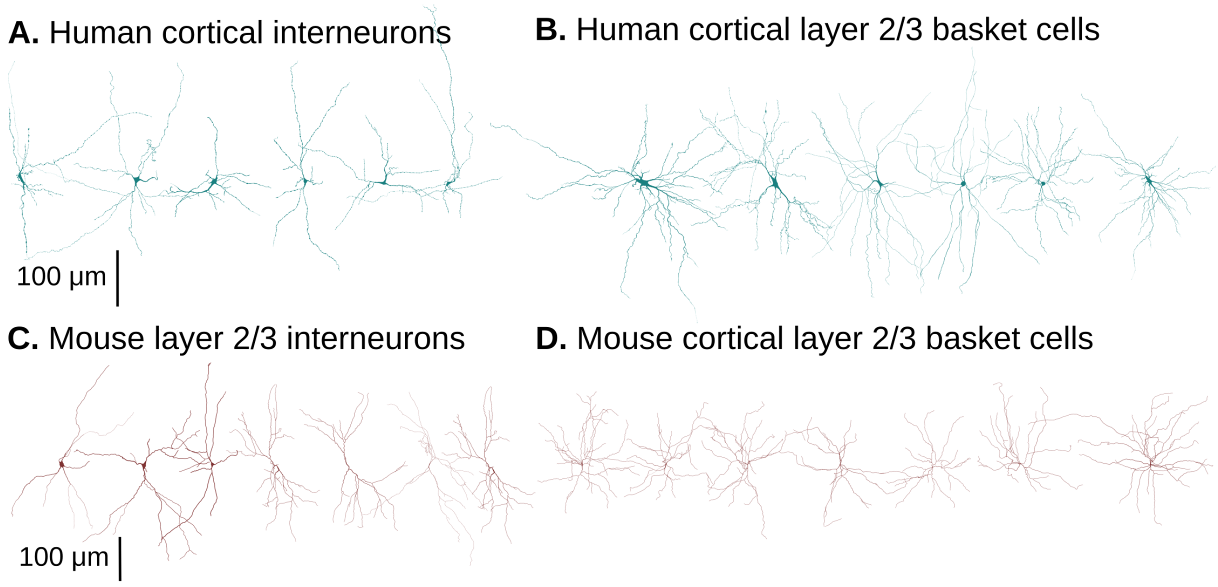

Figure S 8: **Examples of interneuron morphologies.** A. Human cortical interneurons. B. Human layers 2 and 3 basket cells. C. Mouse cortical interneurons. D. Mouse layers 2 and 3 basket cells.

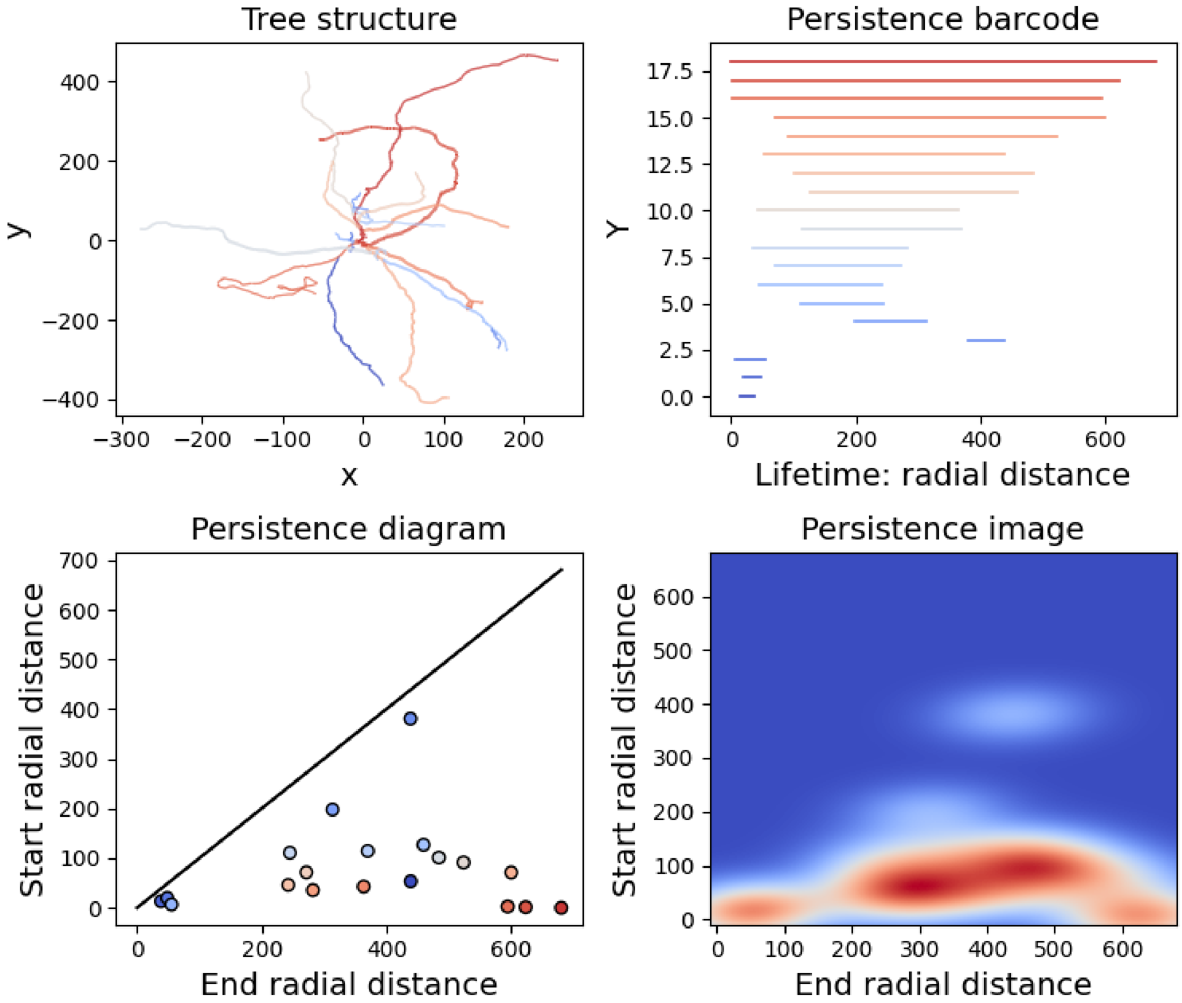

Figure S 9: **Topological morphology descriptor of an exemplar human cortical basket cell dendrites.** A. Basal dendrite, color-coded according to persistence components as illustrated in B. B. Persistence barcode, colormap from largest (red) to smallest branches (blue). C. Persistence diagram with the same color-code. D. Persistence image indicating areas of high density of branches (red) at different path distances from the soma (0,0).

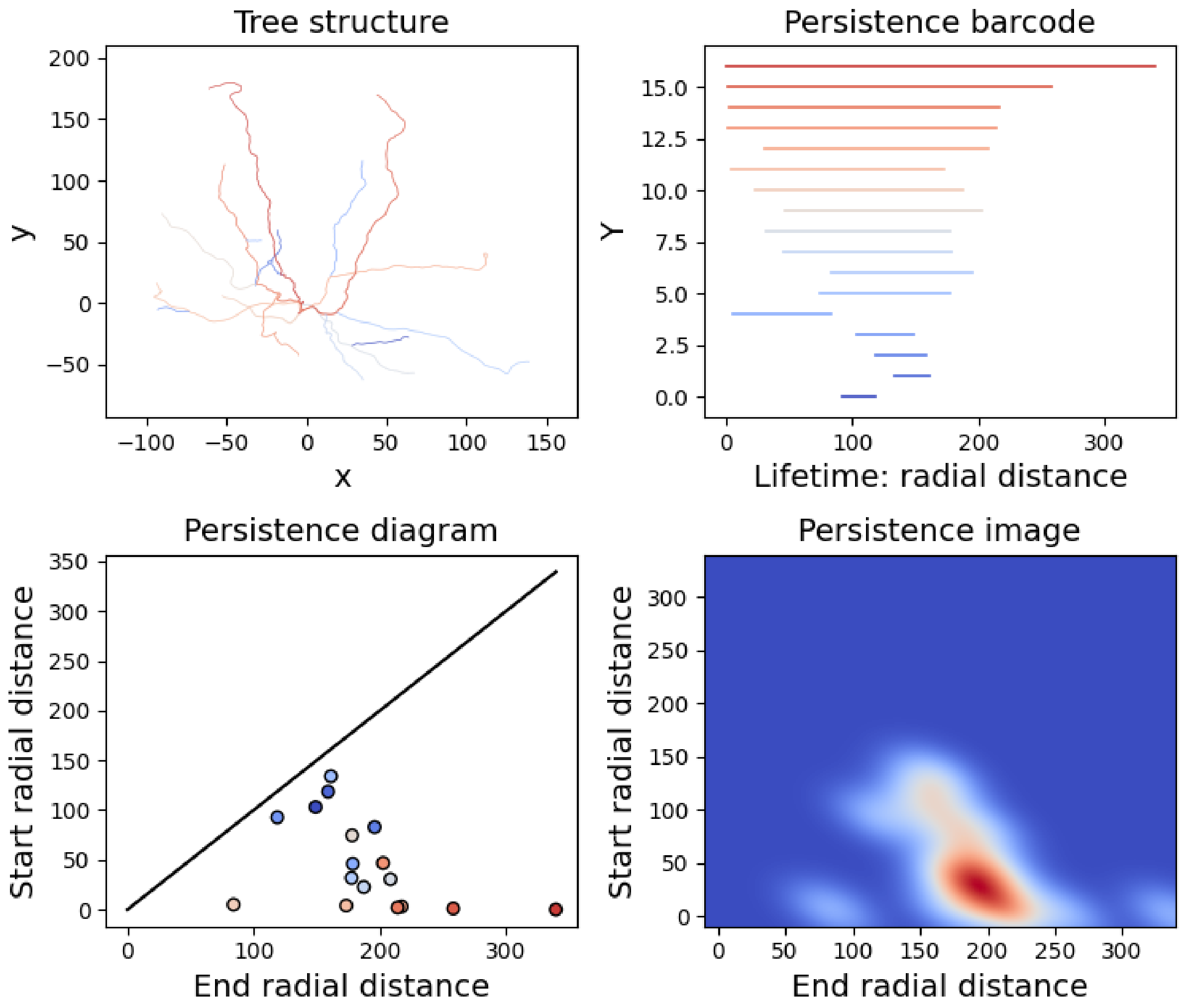

Figure S 10: **Topological morphology descriptor of an exemplar mouse cortical basket cell dendrites.** A. Basal dendrite, color-coded according to persistence components as illustrated in B. B. Persistence barcode, colormap from largest (red) to smallest branches (blue). C. Persistence diagram with the same color code. D. Persistence image indicating areas of high density of branches (red) at different path distances from the soma (0,0).

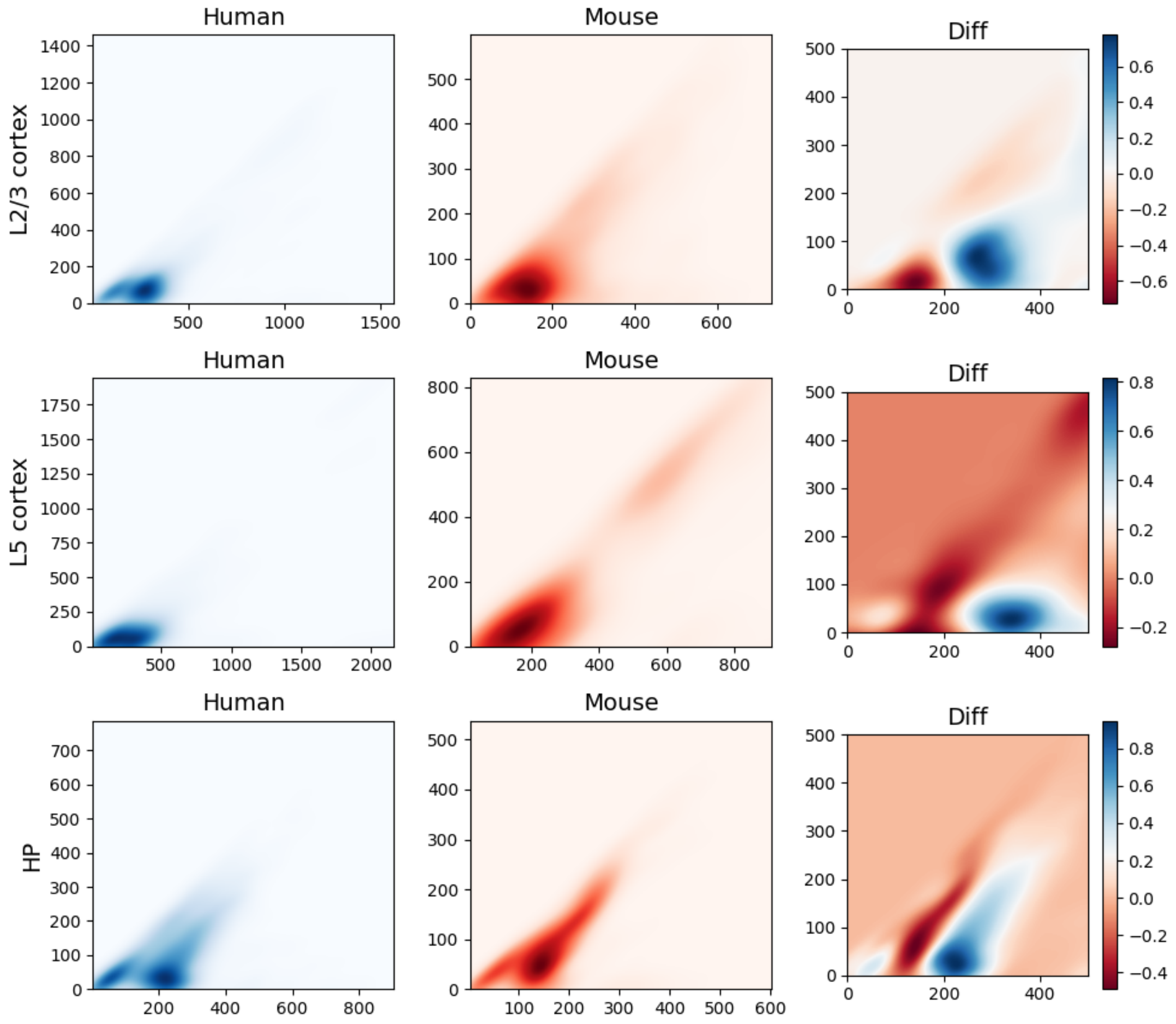

Figure S 11: **Topological analysis of mouse and human pyramidal cells from cortical layers 2, 3, and 5 and hippocampus.** Column 1 shows the average persistence images for populations of human cells from different brain regions in blue. Column 2 shows the average persistence images for populations of mouse cells from different brain regions in red. Column 3 shows the average difference between the persistence images of human (blue) and mouse (red) cells from different brain regions. The topological differences that were observed between human and mouse pyramidal cells of layers 2 and 3 generalize to different layers and brain regions.

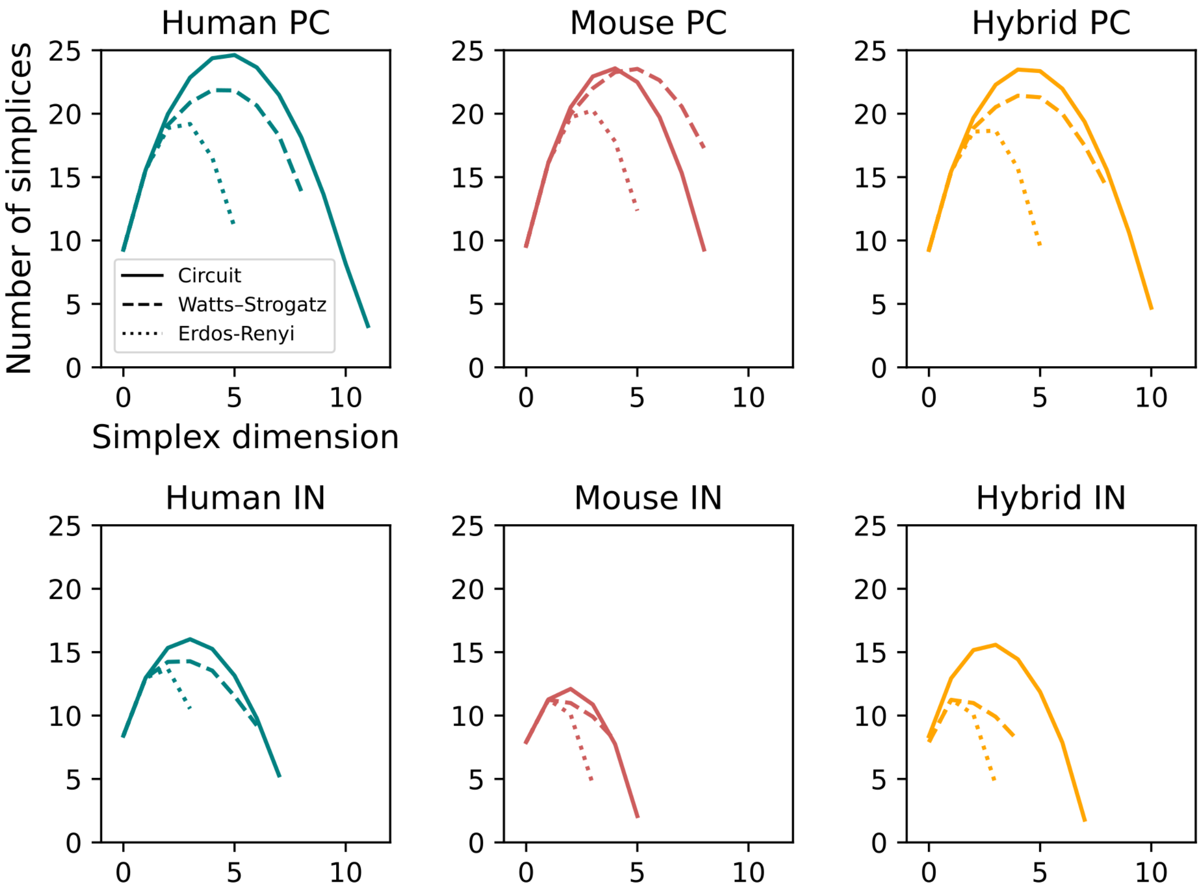

Figure S 12: **Comparison of simplice distribution to random networks.** Simplices of human (pyramidal cells - PC and interneurons - IN), mouse (PC and IN) and hybrid (PC and IN) are compared to random networks based on the Watts-Strogatz and Erdos-Renyi models of the same network size and density.

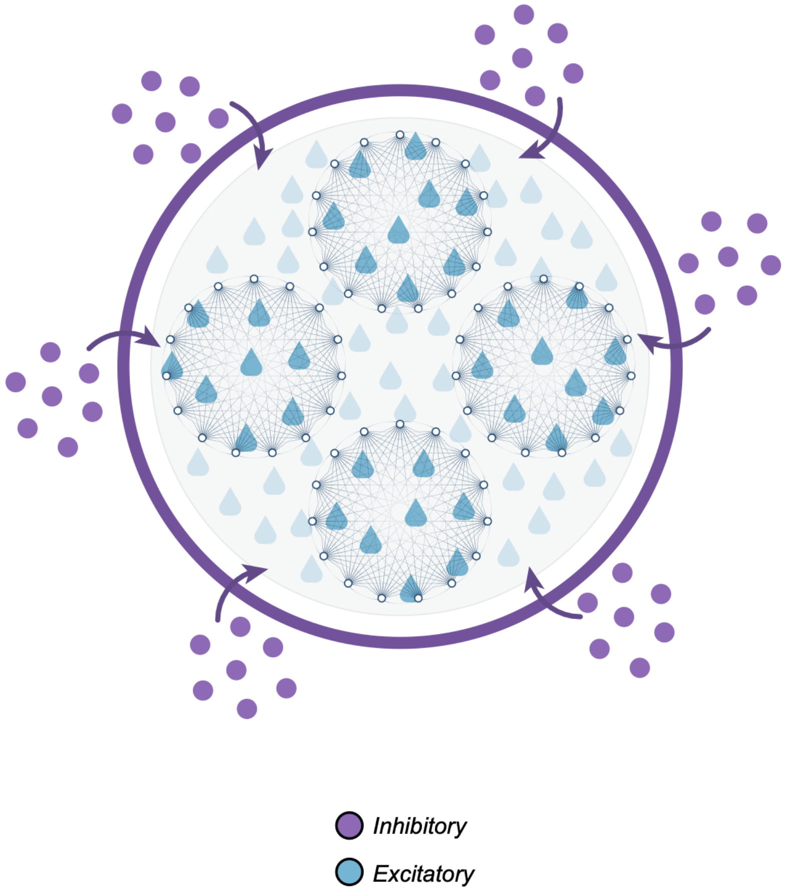

Figure S 13: **Schematic representation of interplay between interneurons and pyramidal cells.** Strongly connected sub-networks of pyramidal cells (cliques) are controlled by small clusters of interneurons. More interneurons are needed to control the highly complex sub-networks of pyramidal cells and balance inhibition-excitation.

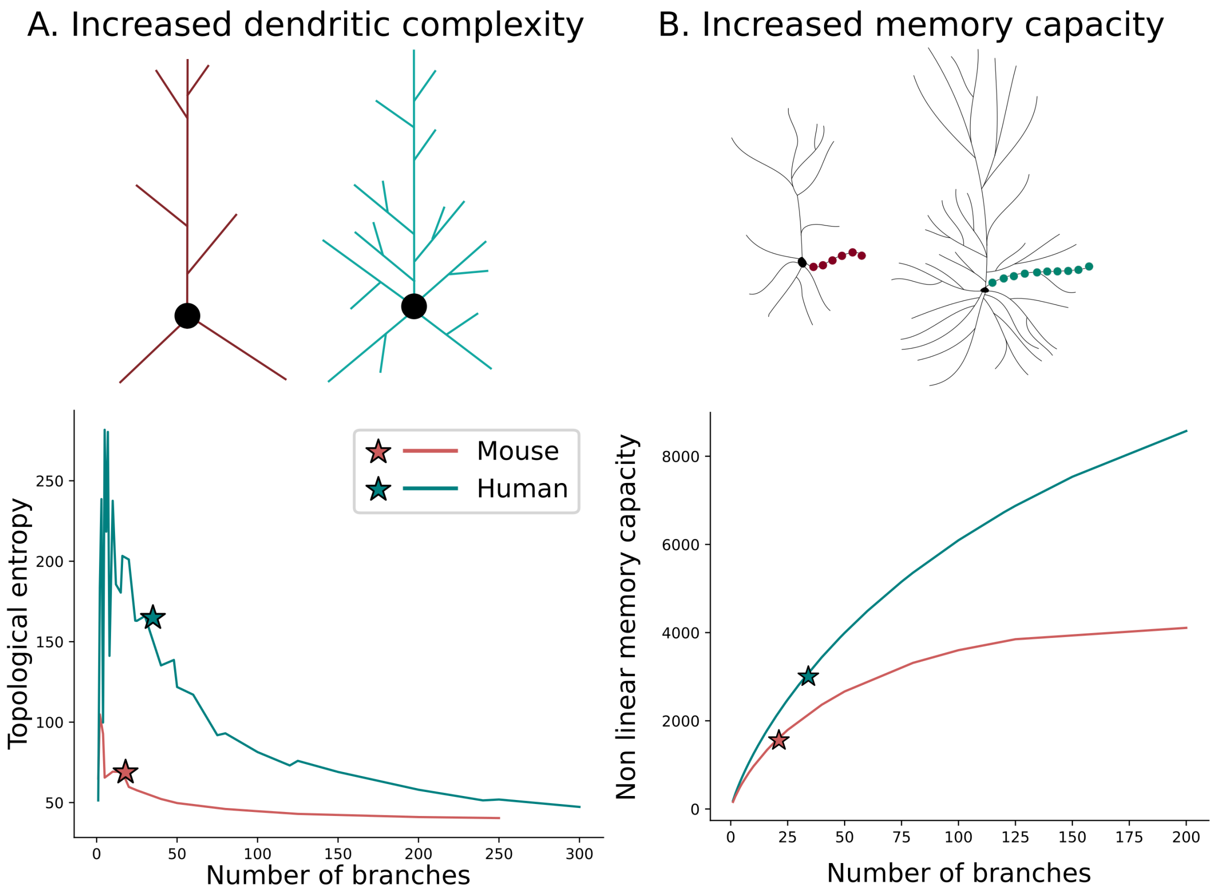

Figure S 14: **Memory capacity is enhanced by cell complexity.** A. Cell complexity is measured by the topological entropy of the dendrites. The stars represent the average values for both species (red: mouse, teal: human). Topological entropy computed based on the average number of branches is 1.8 times higher in human cells. B. Memory capacity is computed by the non-linear formula (see equation (9)) and depends on the number of branches and their lengths. The stars represent the average values for both species (red: mouse, teal: human). Memory capacity computed on the average number of branches is 1.8 times higher in humans.

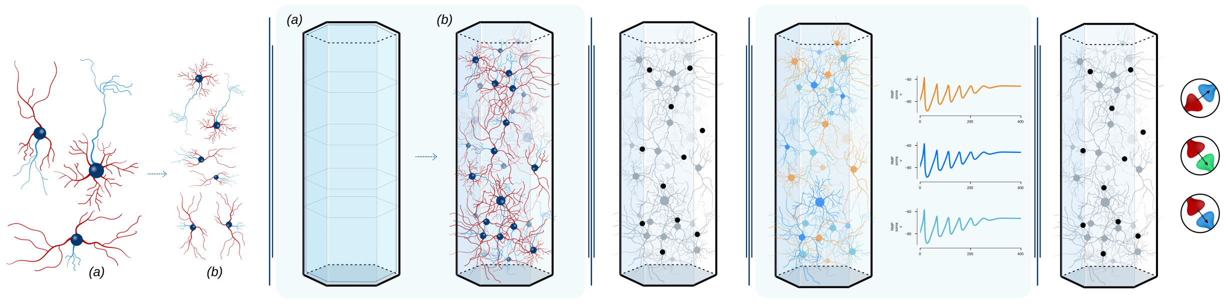

Figure S 15: **Complete pipeline for circuit generation.** In this paper we implement the morphological and connectivity components without functional experiments. A. Morphological diversity. B. Microcircuit anatomy. C. Reconstructing connectivity. Electrical (D) and synaptic (E) diversity are not included in this paper.

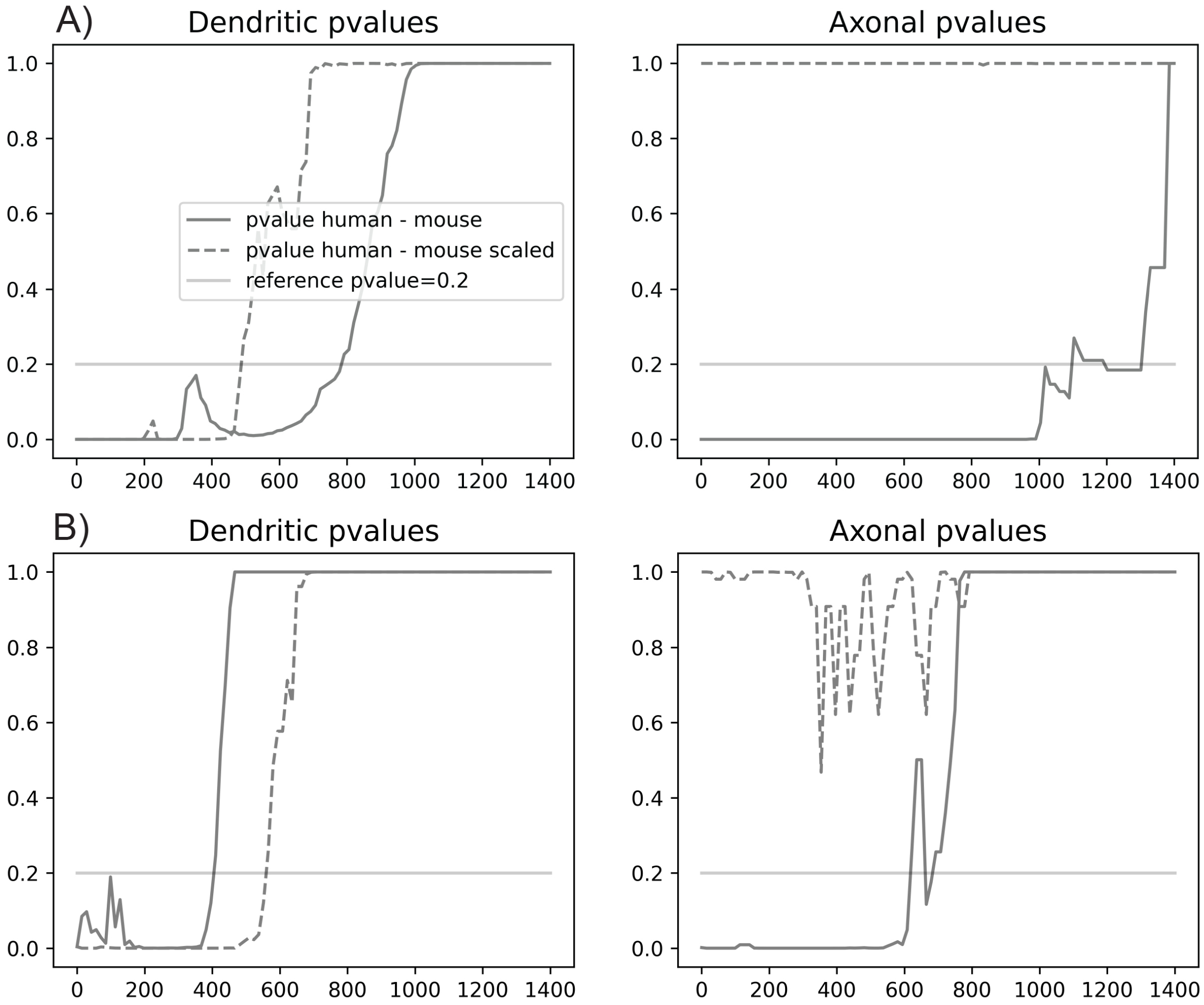

Figure S 16: **Approximation of pvalues for entropy curves.** We approximate the values for the entropy curves by computing p-values at different distances. The dendritic pvalues between human versus mouse and human versus mouse-scaled are presented for pyramidal cell (A) and interneurons (B). The axonal pvalues between human - mouse and human - mouse scaled are presented for pyramidal cell (A) and interneurons (B).
